# An evaluation of the appropriateness of advice and healthcare contacts made following calls to NHS Direct Wales

**DOI:** 10.1186/1472-6963-9-178

**Published:** 2009-09-30

**Authors:** Helen Snooks, Julie Peconi, James Munro, Wai-Yee Cheung, Jaynie Rance, Anne Williams

**Affiliations:** 1 Centre for Health Information, Research and Evaluation (CHIRAL), School of Medicine, Swansea University, Singleton Park, Swansea SA2 8PP, UK; 2 Patient Opinion, 53 Mowbray Street, Sheffield S3 8EN, UK; 3 School of Health Science, Swansea University, Singleton Park, Swansea, SA2 8PP, UK; 4 Nursing, Health & Social Care Research Centre, Cardiff University, Eastgate House, 35 - 43 Newport Road, Cardiff, CF24 0AB, UK

## Abstract

**Background:**

An evaluation of NHS Direct Wales (NHSDW), a national telephone-based healthcare advice and information service, was undertaken. A key objective was to describe the actions of callers and assess the appropriateness of advice and healthcare contacts made following calls, results of which are reported here.

**Methods:**

Postal questionnaires were sent to consecutive callers to NHSDW in May 2002 and February 2004 to determine 1) callers' actions following calls and 2) their views about the appropriateness of: advice given; and when to seek further care. An independent clinical panel agreed and applied a set of rules about healthcare sites where examinations, investigations, treatments and referrals could be obtained. The rules were then applied to the subsequent contacts to healthcare services reported by respondents and actions were classified in terms of whether they had been necessary and sufficient for the care received.

**Results:**

Response rates were similar in each survey: 1033/1897 (54.5%); 606/1204 (50.3%), with 75% reporting contacting NHSDW. In both surveys, nearly half of all callers reported making no further healthcare contact after their call to NHSDW. The most frequent subsequent contacts made were with GPs.

More than four fifths of callers rated the advice given - concerning any further care needed and when to seek it - as appropriate (further care needed: survey 1: 673/729, 82.3%; survey 2: 389/421, 92.4%; when to seek further care - survey 1: 462/555, 83.2%; survey 2: n = 295/346, 85.3%). A similar proportion of cases was also rated through the rule set and backed up by the clinical panel as having taken necessary and sufficient actions following their calls to NHSDW (survey 1: 624/729, 80.6%; survey 2: 362/421, 84.4%), with more unnecessary than insufficient actions identified at each survey (survey 1: unnecessary 132/729, 17.1% versus insufficient 11/729, 1.4%; survey 2: unnecessary 47/421, 11.0% versus insufficient 14/421, 3.3%).

**Conclusion:**

Based on NHSDW caller surveys responses and applying a transparent rule set to caller actions a large majority of subsequent actions were assessed as appropriate, with insufficient contacts particularly infrequent. The challenge for NHSDW is to reduce the number of unnecessary contacts made following calls to the service, whilst maintaining safety.

## Background

In an environment of rising demand for unscheduled care, and increasing options for delivery of care and advice remotely [1,2] twenty four hour health-related telephone advice has been made available to callers across the UK for the cost of a local call. Callers to the NHS funded, nurse-led NHS Direct (NHSD) are offered information, or triaged by nurses, using computer decision software, and directed to emergency, primary or self-care as appropriate.

The service was set up in Wales in 2000 with an explicit focus on triage and an emphasis on promoting self-care or appropriate use of other services, matched to the caller's needs, in order to:

*'help callers by providing the right advice, information and reassurance they require to look after themselves, if appropriate. It will also ensure that callers who need professional help are directed to the right service at the right time... the service is designed to complement traditional primary care services by providing high quality advice and triage to help patients make appropriate use of existing provision of healthcare' *[3].

Research findings published so far concerning user satisfaction of NHSD and provision of opportunities for professional development have been encouraging. NHS Direct's own consistently high caller satisfaction rates (97-99% between March 2005 and February 2006) [4] are matched by independent results (96% of callers reported that the nurse advice they received was quite or very helpful in a postal survey carried out in 1998) [5]. Although a minority of nurses complain that remote nursing can be monotonous and many miss the hands-on role that face to face contact brings, overall the service is seen by nurses working within NHSD and in other parts of the NHS as bringing opportunities for career progression and professional development, in particular through opportunities to gain communication and information technology skills [6-9]. Initial studies have not found evidence of the hoped-for substitution of demand [10,11] and comprehensive information is lacking about what actions callers take following their contact with the service. Call outcomes have been found to vary between sites [11] and between nurses [12-14] but there has been little robust evidence concerning the quality of service in terms of the appropriateness of advice given or safety of the service.

### Measuring appropriateness

Although appropriateness of care has been seen as a fundamental component of quality for some years [15], the slipperiness of the concept of appropriateness is well recognised [16-18] with no agreed 'gold standard' method for measuring the appropriateness of health service procedures or contacts. Although the term is frequently used, the different perspectives of professionals, patients and society at large have been highlighted [17]. Methods for assessing and reporting appropriateness - particularly in emergency medicine - have been the subject of much criticism [19,20]. A wide range of results have been reported across a range of settings [21-25] but it is rare to find a method reported that is transparent and repeatable. Black suggested five areas of concern: dimensions to include in assessment of appropriateness; reliability of methods of assessment of appropriateness; validity of consensus development methods; adaptability of methods to different areas of care; effectiveness of appropriateness of assessment activities in terms of improvements of health care quality or containment of costs [26]. Researchers have found it difficult to avoid relying on clinical assessment or judgement and in the absence of formal criteria for making a decision, agreement between clinicians has been low [27,28]. Although attempts have been made to measure appropriateness of attendance in terms of a categorisation of the processes of care (whether patients have received treatments/investigations/referrals or are admitted, for example), it has proved problematic to achieve consensus about what processes of care make a visit appropriate. Concern continues to be expressed that a more rigorous approach to assessing appropriateness is needed [29].

An evaluation of NHS Direct Wales (NHSDW) was undertaken to assess the effectiveness of the service across clinical, professional and operational dimensions. In this paper we report the results of a strand of the evaluation concerned with assessing the appropriateness of advice received and of healthcare contacts made by patients following calls.

The objectives were to:

• describe the actions of callers following their contact with NHSDW

• assess appropriateness of the service:

◦ in the view of callers

◦ by whether subsequent contacts made to healthcare providers were necessary and sufficient for treatments and investigations carried out

• and to investigate reasons for inappropriate contacts or advice given

## Methods

### Ethical approval and consent

Ethical approval for the study was granted by the Trent Multi Centre Research Ethics Committee. During the study period, when people called NHSDW they were given the opportunity to opt out of participation in research at the beginning of their call and consent to participate specifically in this particular survey was not sought separately.

### Postal questionnaires

Postal questionnaires were sent out to consecutive callers to NHSDW meeting the inclusion criteria in two batches, spaced twenty one months apart, in May 2002 (survey 1) and February 2004 (survey 2). This spacing was to allow for any changes that may have occurred over time and to allow for any seasonal variations in responses. Survey 1 consisted of approximately 2000 callers who had contacted the service during May 2002. Based on the quality and number of responses to this survey, a sample size of 1200 was chosen for survey 2 in order to receive at least 450 responses from people reporting contacting NHSDW.

**Figure 1:**
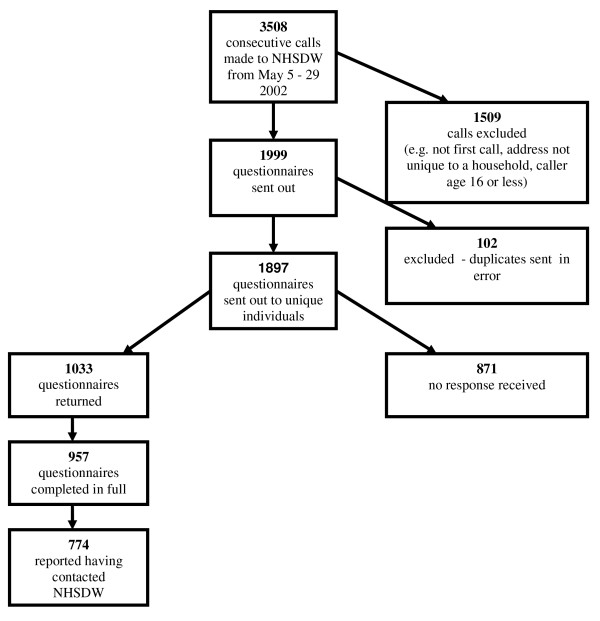
**Flowchart of recruitment selection and responses for survey 1**.

**Figure 2:**
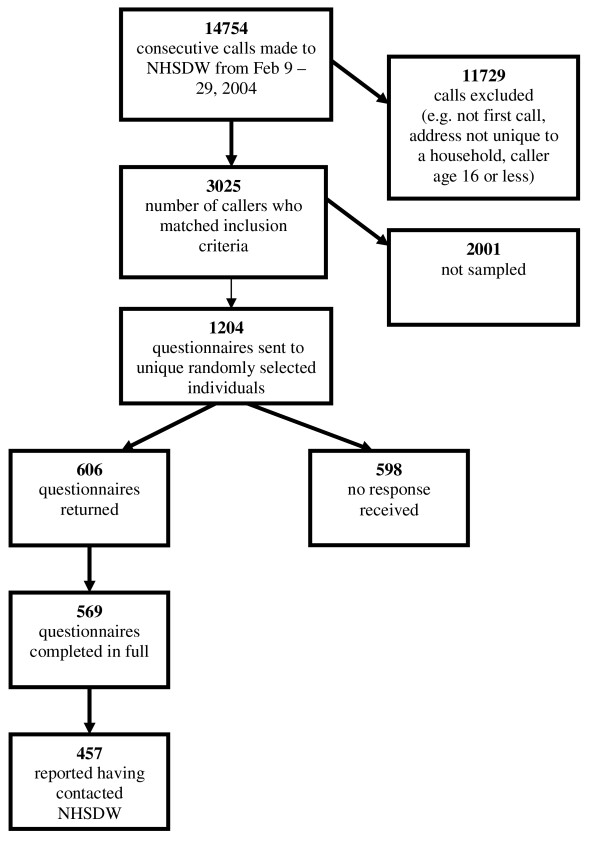
**Flowchart of recruitment selection and responses for survey 2**.

An NHSDW data analyst ran a query to identify to all consecutive, first time callers who met the inclusion criteria within the defined dates. Calls were excluded if the caller was aged less than 16; name and address details were incomplete; the call was not the first one made by the caller during the study period; the address was not unique to a household e.g. business premises, student halls of residence; or if the caller had opted out of participating in research. Figures 1 and 2 outline participant recruitment and response to the study.

The 12 page questionnaire (Additional file 1) related to a contact made in the past eight weeks. It contained questions concerning the call, subsequent healthcare contacts and treatments, investigations and referrals received, and the caller's views about the appropriateness of advice given during the call. Due to concerns about confidentiality and the worry that another member of a household might open the questionnaire which contained an undisclosed contact, the letters were written in general terms and did not reveal that the addressee had called NHSDW. In each survey, two follow-up letters and additional copies of the questionnaire were sent to non-responders.

Data were entered by the study's clerical officer under the supervision of the researcher. A random sample of 10% was checked for accuracy against the raw data.

### Clinical panel rule-based assessment of appropriateness: approach taken

In this study, we used a method of assessing appropriateness [30] that addresses the aforementioned concerns with processes of care. The concepts of 'necessary' and 'sufficient' were used as an indicator of appropriateness and applied to patient's reported actions following their call with NHSDW (e.g. the healthcare contacts they made and the resulting treatments). Healthcare contacts made following calls to NHSDW were then deemed to be appropriate if the patient needed to attend that particular level of service in order to access the care received. In other words, the care they received was both necessary and sufficient. In order to provide a balance to this clinically derived process-based assessment, we also report the views of service users about the appropriateness of advice given.

Using this method, the quality of advice was not directly assessed, but rather the appropriateness of the outcome of the call - the action taken by the caller as reported in the postal questionnaires. From the care the patient received it was judged whether the patient's contact with the service was 1) necessary: could the patient have been seen elsewhere to have received this care? and 2) sufficient: did the patient need to make a further healthcare contact to receive the level of care required? This method relies on a number of central assumptions:

1. That a "hierarchy of care" can be defined. For the purposes of this study, the hierarchy of care was defined (from top to bottom) as Accident and Emergency (A&E) department, general practice, nurse-led services (such as minor injury units and walk-in centres), community pharmacy services, and finally self-care.

2. That patients should normally be directed to the "lowest" level of care able to meet their needs.

3. That services are assumed to do all that is necessary (including referral, if need be) and nothing which is not necessary, to meet the patient's needs.

4. That a patient will seek further help if their problem remains unresolved or worsens.

5. That all relevant processes of care can be recognised and reported by patients.

Processes of care (events, treatments, examinations) which a patient might report as having occurred at a service were listed and matched to service levels in a rule set (Table 1) by a clinical panel convened for the study, including specialists in emergency medicine (n = 2, both with extensive clinical research experience), general practice (n = 2, one also a medical director of NHSD, the other with previous management in a GP co-operative, a walk-in centre and an NHSD site), and telephone-based nursing (n = 2 NHSD employees, one nurse and one manager). Clinical panel members were recommended by members of the study team and were recruited through invitation letters, followed up by visits from the study lead and researcher to explain the process in more depth.

**Table 1:** Clinical panel rule set

**Event statement**	**The lowest level of care at which this process/intervention can reasonably be expected to be provided**
No physical exam	Self care
Not sure if physical exam	Self care
Chest examined	GP or emergency doctor
Abdomen examined	GP or emergency doctor
Skin examined	MIU/WIC*
Eyes examined	GP or emergency doctor
Ears, nose or mouth examined	MIU/WIC
PR or PV exam	GP or emergency doctor
Limbs examined	MIU/WIC
Other examination was done: (free text answer)	Requires expert review
No tests	Self care
Not sure if any tests were done	Self care
Blood tests were done	GP or emergency doctor
An ECG was done	GP or emergency doctor
X-rays/scans were done	A&E
Urine tests were done	MIU/WIC
Other tests were done: (free text answer)	Requires expert review
No treatment	Self care
Not sure if treatment	Self care
Drip or injection there and then	Self care
The drip or injection was:	Requires expert review
Medicine or prescription to take later	Pharmacist
The medicine or prescription was:	Requires expert review
A dressing, sling or support bandage was provided	MIU/WIC
Plaster was applied	A&E
Stitches were used to close a wound	MIU/WIC
Steristrips were used to close a wound	MIU/WIC
Glue was used to close a wound	MIU/WIC
Other treatment was given: (free text answer)	Requires expert review
Advice/information was given	Self care
Advice:	Requires expert review
Follow up appointment was made	A&E
They said contact was right	
They said contact was not right	
No comment on contact	
Uncertain if comment on contact	
Comment on contact was:	Requires expert review
No further care was advised/arranged	Self care
Not sure about further care	Self care
Hospital admission was arranged	A&E
A&E was advised	GP or emergency doctor
MIU/WIC was advised	Pharmacist
Hospital clinic was arranged	GP or emergency doctor
Advised to see GP	Pharmacist
Advised to see pharmacist	Self care
Advised to see a dentist	Pharmacist
Other care was advised/arranged	Self care
Other care advised or arranged:	Requires expert review
A GSL medication was provided	Self care
A pharmacy medication was used	Pharmacist
A nurse-prescribable medication was used	MIU/WIC*
A doctor-prescribable medication was used	GP or emergency doctor
An IV infusion was used	A&E
A stat IV or IM drug was given	GP or emergency doctor
An A&E prescribable treatment was used	A&E
Back examination	GP or emergency doctor
Ribs examination	GP or emergency doctor
Blood pressure examination	MIU/WIC
Pulse/heart rate examination	MIU/WIC
Breast examination	GP or emergency doctor
Conscious level examination	MIU/WIC
Fontanelle examination	GP or emergency doctor
Head injury examination	MIU/WIC
Face examination	GP or emergency doctor
Gums examination	MIU/WIC
Neck examination	GP or emergency doctor
Shoulder examination	GP or emergency doctor
Temperature measured	MIU/WIC
Reflexes	GP or emergency doctor
Blood oxygen test	A&E
Blood sugar test (BM stix)	MIU/WIC
Stool test	GP or emergency doctor
Heart rate monitor	A&E
CT scan	A&E
Gas and air	MIU/WIC
Nebuliser	GP or emergency doctor
Oxygen	A&E
Nose cauterised	A&E
Nail trephine	MIU/WIC
Eye wash for trauma	A&E

*MIU = Minor Injuries Unit, WIC = Walk in Clinic

Based on the agreed rule set, a computer algorithm was constructed and survey responses were processed. Cases which could not be unambiguously categorised by the computer alone (e.g. those where the category depended on a free text response) were referred to the clinical panel for assessment, again, applying the rule set. To rate cases, panel members were divided into partners and each given a set of cases. Members with different backgrounds were paired together and each person was asked to rate and submit their cases individually. Results were tabulated and in cases in which both partners agreed, this became the rating. When disagreement occurred, partners were asked to discuss the case and if they could reach an agreement, a rating was determined. If after discussion no agreement was reached, the case was then presented before the whole clinical panel and a discussion ensued.

As a result, for all survey responses, the rule set was applied, either by computer or by clinician, to the processes of care reported.

Thus all contacts were categorised, initially by computer algorithm and backed up by a clinical panel, as:

• necessary and sufficient

• insufficient (although necessary)

or

• unnecessary.

In order to link this assessment to advice given during calls, transcripts of taped calls from survey 1 were reviewed. Insufficient contacts could indicate unsafe care and were expected to be a relatively infrequent occurrence, therefore all calls for which subsequent contacts were assessed as insufficient were reviewed. Unnecessary contacts represent less serious - although important - potential inefficient resource usage as well as inconvenience for patients. Since these events are expected to occur more often, a random sample of those who had made contacts that were assessed as unnecessary were retrieved from NHSDW for review. All panel members were presented with the transcripts, a summary sheet with details of actions the caller had reported taking following their call and rating sheets for assessment. Panel members were asked to rate each case according to whether they felt that the advice given was clinically justifiable or explicable for some other reason, and to provide comments.

### Analysis

User survey questionnaire responses were entered into an Access database and transferred to SPSS Version 12.0 for analysis. Data were analysed using descriptive frequencies and, for comparisons between groups, chi squared tests. Data from the panel rating of transcripts were analysed descriptively and are presented in summary form.

## Results

### Completeness of data

1033/1897 (54.5%) callers returned their questionnaires at the first survey, with 957 sufficiently completed to be usable (Figure 1). The response rate at the second survey was slightly lower, at 50.3% (606/1204), with 569 usable (Figure 2).

For both surveys, no significant differences were found in the call type recorded by NHSDW or gender between respondents and non-respondents, and the profile of advice given (disposition) was remarkably similar (Table 2). In both surveys, respondents were a little older than non-respondents (survey 1: 46.3 years vs 39.8; survey 2: 46.8 years vs 38.7, p < 0.001).

**Table 2:** Comparison of the characteristics of respondents and non-respondents

		**Survey 1**			**Survey 2**		
		**Respondents**	**Non-Respondents**	**p**	**Respondents**	**Non-Respondents**	**p**
***NHSDW Call Type***							
Direct calls:	Triage	572 (60.4)	580 (61.2)	0.37	311 (54.7)	372 (58.6)	0.16
	General information	267 (28.2)	248 (26.2)		216 (38.0)	206 (32.4)	
Indirect calls:	GP out of hours	103 (10.9)	114 (12.0)		25 (4.4)	26 (4.1)	
	A&E/Ambulance	5 (0.5)	5 (0.5)		3 (0.5)	8 (1.3)	
	Dental helpline	0 (0.0)	0 (0.0)		14 (2.4)	23 (3.6)	
	Missing/Misdirected	3	0 (0.0)		0 (0.0)	0 (0.0)	
	**Total**	950* (100.0)	**947 (100.0)**		**569 (100.0)**	**635 (100.0)**	
***NHSDW Dispositions***							
	999/A&E	75 (7.9)	91 (9.6)	0.54	54 (9.5)	58 (9.1)	0.68
	Emergency GP	237 (24.9)	231 (24.4)		91 (16.0)	96 (15.1)	
	GP	208 (21.9)	177 (18.7)		94 (16.5)	107 (16.9)	
	Dentist	15 (1.6)	20 (2.1)		28 (4.9)	51 (8.0)	
	Other	13 (1.4)	31 (3.3)		16 (2.8)	22 (3.5)	
	Pharmacist	7 (0.7)	3 (0.3)		6 (1.1)	6 (0.9)	
	Dentist information	0 (0.0)	0 (0.0)		117 (20.6)	128 (20.2)	
	Home care/Information	355 (37.4)	356 (37.6)		141 (24.8)	144 (22.7)	
	Unclassifiable	40 (4.2)	38 (4.0)		22 (3.9)	23 (3.6)	
	**Total**	**950 (100.0)**	**947 (100.0)**		**569 (100.0)**	**635 (100.0)**	
***Gender***							
	Male	262 (27.6)	295 (31.2)	0.08	192 (33.7)	216 (34.1)	0.95
	Female	688 (72.4)	650 (68.8)		377 (66.3)	418 (65.9)	
	Missing	0 (0.0)	2		0 (0.0)	1	
	**Total**	**950 (100.0)**	**947 (100.0)**		**569 (100.0)**	**635 (100.0)**	
*7 returned questionnaires were missing a call id and as a result, although they were useable questionnaires bringing the total number up to 957, they have been included here as non respondents as they could not be identified to remove.							

*7 returned questionnaires were missing a call id and as a result, although they were useable questionnaires bringing the total number up to 957, they have been included here as non respondents as they could not be identified to remove.

At each survey, NHSDW data showed that respondents had called NHSDW themselves for healthcare information or advice (survey 1: 73.8%, n = 706; survey 2: 74.9%, n = 426). A smaller proportion reported having been transferred through from their GP out of hours service, (survey 1: 14.3%, n = 68; survey 2: 5.4%, n = 31).

A significant proportion of respondents denied having used the service (survey 1: 14.3%, n = 137; survey 2: 19.7%, n = 112) while 46 respondents in survey 1 did not indicate how they came to be in touch with NHSD. For those who denied contact with the service, there did not appear to be any apparent patterns with regards to age or gender.

All further analysis was restricted to those 774 patients from survey 1 and 457 patients from survey 2 who indicated that they called NHSDW in the previous eight weeks.

### Demographics

For survey 1, the mean age of respondents was 45.0 years and 73.9% were female. For survey 2, the mean age of respondents was 46.8 years and 66.3% were female. There was a spread of respondents across age groups, particularly between 20 and 70 years.

### Actions of callers after contacting NHSDW

**Figure 3:**
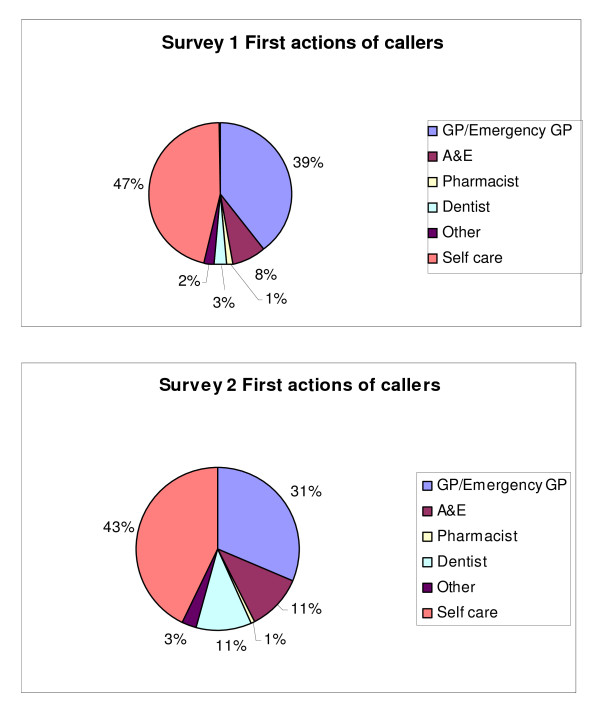
**First actions by callers after calling NHSDW**.

At each survey, just over half of all respondents reported going on to contact a further service, with a few making more than one subsequent contact. In both surveys, the services contacted most frequently following the call to NHSDW were GPs/Emergency doctors, dentists and A&E departments (Figure 3). In survey 1, no one reported re-contacting NHSDW for additional information after their original call, while in survey 2 two callers reported contacting NHSDW again, as their second contact following their initial call (however in this paper we are concerned only with the first contact after the call).

### Assessment of appropriateness

Over 90% of respondents at each survey reported that the advice given about care and when to seek further help was quite or very appropriate, helpful and easy to follow (Table 3). The rule based rating of appropriateness also assessed a large majority of cases as having made contacts that were both necessary and sufficient, with more calls assessed as having resulted in unnecessary contacts than insufficient contacts at each survey (Table 3).

**Table 3:** Callers' views about advice given during their call to NHSDW and rule based rating

	**Survey 1**		**Survey 2**	
	**n**	**%**	**n**	**%**
***Appropriateness of advice***				
Very appropriate	500	68.6	302	71.7
Quite appropriate	173	23.7	67	20.7
Not very appropriate	27	3.5	17	4.0
Not appropriate at all	29	3.7	15	3.6
Not answered	45		36	
				
***Appropriateness of advice about timing***				
About right	462	83.2	295	85.3
Problem was more urgent than NHSD said	15	2.7	7	2.0
Problem was less urgent than NHSD said	20	3.6	9	2.6
No advice given on timing	58	10.5	35	10.1
Not answered	219		111	
				
***Helpfulness of advice***				
Very helpful	495	69.1	297	71.6
Quite helpful	163	22.8	82	19.8
Not very helpful	39	5.5	22	5.3
Not helpful at all	19	2.7	14	3.4
Not answered	58		42	
				
***How easy it was to follow advice***				
Very easy	541	76.7	324	76.8
Quite easy	119	16.9	62	14.7
Quite difficult	32	4.5	16	3.8
Very difficult	13	1.8	20	4.7
Not answered	69		35	
				
**Total**	**774**	**100.0**	**457**	**100.0**
				
***Rule based rating of health actions following call***				
Necessary and sufficient	624	80.6	362	84.4
Unnecessary	132	17.1	47	11.0
Insufficient	11	1.4	14	3.3
Unclassifiable	7	0.9	6	1.4
**Total**	**774**	**100.0**	429*	**100.0**

*dental calls were excluded as shortages in provision in Wales at the time of survey 2 distorted the assessment of appropriateness of contacts made for these calls

For those callers who were assessed as having taken unnecessary actions following their call, the majority of unnecessary actions concerned contacting a GP (survey 1: 71%, n = 94; survey 2: 68%, n = 32) or visiting the A&E Department (survey 1: 22%, n = 29; survey 2: 21%, n = 10). A few other callers went on to the dentist (survey 1: 5%, n = 6; survey 2: 9%, n = 4), or made other healthcare contacts. Similarly, the majority of callers assessed to have taken insufficient action following their call to NHSDW made contact with a GP (survey 1: 55%, n = 6; survey 2: 64%, n = 9), with a minority having contacted a dentist, hospital clinic, pharmacist or Minor Injuries Unit.

Taped calls were retrieved from survey 1, for eight of the 11 cases (the other three calls could not be retrieved) where subsequent contacts were judged to have been insufficient and for 15 of 26 sampled cases where subsequent contacts were judged to be unnecessary. These were transcribed and reviewed. NHSDW were unable to match the data for the remaining calls from the information given by the respondent.

Results of the review are summarised in Additional File 2: Table S1. Of the eight cases highlighted by the rule based assessment as 'insufficient subsequent action', three were also supported as 'insufficient subsequent action' by at least two panel members in each case. In these cases (639, 884, 2505) there was a lack of consensus between panel members ('insufficient' ratings ranged from 2-3 members), although concerns were raised that the advice given was inappropriate and had led to the insufficient action taken. In all of these cases, callers rated the advice given as 'very appropriate'. In a further three cases the clinical panel members agreed that the advice given was clinically justifiable and in a further two was explicable in some other way.

Of the 15 cases assessed through the rule based process as 'unnecessary subsequent action', the clinical panel members agreed that the advice given was clinically justifiable in nine cases. In three other cases (1193, 961, 651) the rule based method assessment was overturned on review of the transcript when at least one panel member judged that the advice given was insufficient for the problem. In three further cases a minority of panel members rated the advice to contact an emergency GP as unnecessary, although the other panel members assessed the advice as clinically justifiable (648, 419, 53). Of these cases (n = 6) in which clinical panel members judged the advice given to be either insufficient or unnecessary, callers rated the advice as 'very appropriate' (n = 5) or 'quite appropriate (n = 1).

From comments and variation in judgements made, the panel members appear to have found this process difficult to carry out. However although there was a lack of consensus in many cases, there were some overall messages:

1. In most cases the clinical panel members agreed that the advice given by NHSDW nurse advisors was clinically justifiable and appropriate

2. In a minority of cases concerns were raised that advice given led to insufficient actions

3. In a further small minority of cases, advice given was judged to have led to unnecessary actions

## Discussion

Questionnaire response rates in this study were 54.5% (survey 1) and 50.3% (survey 2). In each survey, just over half of respondents reporting going on to contact further services -most frequently the GP or emergency GP- after their initial call to NHSDW. The majority of respondents at each survey reported the advice they received to be appropriate, helpful and easy to follow. The rule based rating of appropriateness also assessed a large majority of cases as having made contacts that were both necessary and sufficient. More calls were judged as having resulted in contacts which were unnecessary rather than insufficient. Of the sample of calls in which transcripts underwent clinical panel review, in most cases members agreed that the NHSDW advice was clinically justifiable and appropriate, although concerns were raised that advice given had led to insufficient or unnecessary actions in a small minority of cases.

Both approaches to the assessment of appropriateness made in this study - based on the view of the caller, or the rule based assessment - rested on the self reported perceptions and actions of callers who responded to the questionnaire. Although attempts were made to increase response rates including sending out additional copies of the questionnaire and attaching letters to the recipients, with a response rate of just over 50%, it is important to acknowledge that the other 50% may have had different experiences of care, may have taken different actions following their calls and may have held different views about the quality of care they received. Within the resource constraints of the study, we were also unable to verify accounts of contacts made and treatments received, but acknowledge this as a potential area of inaccuracy. Additionally, problems with retrieving taped calls meant the full sample of calls where actions were judged to be clinically inappropriate could not be analysed to understand possible explanations.

The assumptions that underlie the rule based assessment of whether actions taken were necessary and sufficient can be challenged - and were, quite frequently, by the clinical panel members. In particular, the assumption that treatments and investigations received were 'de facto' necessary was quite difficult for the panel members to accept. Indeed, there may be a tendency for patients to receive treatments and investigations that are available at the place of care that they attend, but that they may not be referred on for them if they attend a place of care where the services are unavailable. If this is the case, this would have the effect of artificially inflating the rate of appropriateness reported in this study. The rate of unnecessary contacts would likewise be artificially low, but the rate of insufficient contacts may be less likely to be influenced by this artefact.

The clinical panel assessment of cases where contacts were judged to have been inappropriate was a challenging process. Lessons can be taken forward from this study concerning the need to use a formal process of achieving consensus as well as the resources required to undertake this work and achieving a balance of clinical panel membership across disciplines and areas of expertise. In the future, consideration could be given to other research methods including case studies, participant interviews or observation.

Methodologically, whilst not perfect this study has met its objectives by using an approach that is transparent and repeatable. Findings reported here rest on explicit assumptions, rather than the implicit foundations of clinical judgement and are therefore open to interpretation and adjustment as suited to the context of application. The method used now needs to be validated through further application in other settings and sites, but will then meet Lowe et al's 2001 quality standards for appropriateness research in emergency care, regarding definition, societal context and implicit assumptions [20].

Appropriateness, alongside technical competency and human dignity has been seen as one of the fundamental aspects of quality of care. Although a widely used (RAND corporation) definition rests simply on whether the benefits of any procedure or service outweigh any risks by a wide enough margin to make it worthwhile providing [18], this definition is so broad that interpretation of appropriateness varies widely between assessor - even within professional group - and importantly, in ways that are not transparent [31]. Naylor suggests that what is assessed as appropriate care depends on who is asked; their location; the weight given to different types of evidence and outcomes; whether service user preferences are considered; the level of resources available; and prevailing societal and system values [31]. In the case of NHSD, a new service that provides information, advice and signposting, the appropriateness of outcomes achieved seems to be critical to any assessment of effectiveness.

In an attempt to overcome previous difficulties in this field and to reduce variation in assessments, we used an approach that matched processes of care - the place (and level) of health contact made and what was done there: treatments, investigations, advice, and referral. By gathering the views of service users alongside this clinically derived process based assessment, we hope to have reported a balanced picture of the appropriateness of advice given by the service and of its outcomes.

## Conclusion

We have described comprehensively the actions of callers following calls to NHSDW and assessed appropriateness in the view of callers and from their subsequent healthcare contacts. We have also assessed clinical appropriateness of advice given to callers whose actions were deemed inappropriate. With most callers going on to manage their condition on their own or contact their GP, and most contacts rated as appropriate through the rule based assessment, this study has not raised major concerns related to safety or wasteful practice. However, with a conservative estimate of one in six calls resulting in a further unnecessary health care contact irrespective of whether the callers are doing as advised by NHSDW, there is no room for complacency. Across Wales, with 311,407 calls received by NHSD in 2003/4, this would translate to more than 53,000 associated subsequent healthcare contacts that were not necessary in terms of treatments or investigations received. The challenge for NHSDW now appears to be to reduce unnecessary contacts whilst maintaining safety, as evident in the current low undertriage ('insufficient contact') rate.

## Competing interests

The authors declare that they have no competing interests.

## Authors' contributions

HS was the Principal Investigator of the NHSDW Evaluation Project. She oversaw data collection, contributed to analysis and interpretation and was responsible for the first draft of this paper. JM devised the clinical panel method of measuring appropriateness and was responsible for data analysis for this section. WYC provided statistical input and data analysis. JP organised the clinical panel for the study and was responsible for subsequent drafts of this manuscript. JP and JR were responsible for data collection and contributed to data analysis and interpretation. AW participated in study design and data analysis. All authors provided substantial comments to earlier drafts of this manuscript and have read and approved the final manuscript.

## Pre-publication history

The pre-publication history for this paper can be accessed here:



## Supplementary Material

Additional file 1 **Health Care Survey for the NHS**. This document is the questionnaire sent to callers to NHSDW. Click here for file

Additional file 2 **Table S1: Transcript review: summary of cases, ratings and comments**. This table contains clinical panel ratings and comments. Click here for file
