# Supplementary material for: An evaluation of the appropriateness of advice and healthcare contacts made following calls to NHS Direct Wales
Source: BMC Health Serv Res. 2009 Sep 30;9:178. doi: 10.1186/1472-6963-9-178 (PMC2761899; doi:10.1186/1472-6963-9-178)
Supplement: Additional file 1 — Health Care Survey for the NHS. This document is the questionnaire sent to callers to NHSDW. [file 1472-6963-9-178-S1.DOC]

# CONFIDENTIAL

| 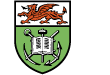 | The Clinical SchoolUniversity of Wales Swansea |
| --- | --- |
|  |  |

**Health Care Survey for the NHS**

This survey is being carried out for the NHS. It asks you about recent use of health services.

Please give an answer to every question. The information you provide will be very valuable in helping to plan future health services, even if you feel the survey doesn’t apply directly to you.

Completing the survey will probably take you 5 to 10 minutes. Your name and address do not appear on this booklet and the information you give will not be presented in any way that could identify you.

An envelope has been provided for your reply, and you don’t need a stamp.

The questions which follow are for **the person whose name appears on the envelope**.

If that person cannot fill in the answers for themselves and you are doing it for them, please remember to give answers for the person named on the envelope, and not for yourself.

**1. How old are you? years**

2. Are you male ❑ female ❑

3. Are you registered with a family doctor (GP)?

Yes ❑

No ❑

Not sure ❑

**4. Which of the following apply to you:**

I am in full time employment ❑

I am in part time employment ❑

I am unemployed……………………...❑

I am a housewife / house-husband… ❑

I am a student………………………… ❑

**5. If you are in paid employment, what is your occupation?**

6. In the last 8 weeks, have you contacted NHS Direct Wales for advice or information?

Think about any contact you made yourself or someone else made for you

| Yes, I called NHS Direct Wales myself, or  someone called for me ❑ | **If yes,** please continue below |
| --- | --- |
| Yes, I tried to contact my own doctor  but was put in touch with NHS Direct Wales ❑ | **If yes,** please continue below |
| No, I haven’t spoken to NHS Direct Wales ❑ | **If no,** you do not need to answer any more questions. Please return this questionnaire in the envelope provided |

About your last contact with NHS Direct Wales

If you have spoken to NHS Direct Wales more than once, please just think about the **last** contact you made

**7. When did you last contact NHS Direct Wales?** Give your best guess if you are not sure

Date for example, 29 January 2002

Time am/pm for example, 11:30 am

8. Thinking just about this last call, what sort of problem was it that you called about? (For example, sore throat, vomiting, back pain)

9. What advice did NHS Direct Wales give you?

Please tick all the options that were suggested

They put me through to the 999 emergency service ❑

They advised me to go to:

a hospital casualty department ❑

a minor injury unit or a walk-in centre ❑

They advised me to contact

my doctor ❑

an emergency doctor (GP) ❑

a pharmacist ❑

a dentist ❑

They told me how to manage the problem myself ❑

Other advice: please say what

I’m not sure ❑

10. If NHS Direct Wales advised you to contact another service, how soon did they say you should do this?

| Immediately ❑  The same day ❑ | The next day ❑  Not sure ❑ |
| --- | --- |

If other, please say how soon

11. Did you act on the advice?

| Yes, all of it ❑  Yes, some of it ❑ | No, none of it ❑ |
| --- | --- |

If there was any advice you did not act on, please say why

12. How easy was it to follow the advice in practice?

| Very easy ❑  Quite easy ❑ | Quite difficult ❑  Very difficult ❑ |
| --- | --- |

If you found it difficult, please say why

After your call

**13. After your call, did you in fact contact any services to get more help for this problem?**

Over the next few hours or days did you contact your doctor, the hospital, a pharmacist, or any other service, **whether or not** NHS Direct Wales had advised it?

No, I looked after the problem myself ❑ **If no,** please go straight to question 31⮚

Yes, I did contact another service ❑ **If yes,** please continue below

**14. Please list the names of all the services you contacted, starting with the first one**.

|  | **Name** and address of service | **Type** of service | **When** contacted  Give the **time and date** or say **how long after** your call |
| --- | --- | --- | --- |
| Example | Dr Smith 51 High St, Anytown | My own doctor | 10am on 12 June  (12 hours later) |
| **First** I contacted...  Was this contact:  By telephone only?….. ❑ OR face to face?….. ❑  If face to face:  Did the GP call to see you at home?….. ❑ OR did you go to the surgery?….. ❑  If you went to the surgery, how far did you travel?………………………………….. | | | |
| And then I contacted…  Was this contact:  By telephone only?….. ❑ OR face to face?….. ❑  If face to face:  Did the GP call to see you at home?….. ❑ OR did you go to the surgery?….. ❑  If you went to the surgery, how far did you travel?………………………………….. | | | |
| And then I contacted…  Was this contact:  By telephone only?….. ❑ OR face to face?….. ❑  If face to face:  Did the GP call to see you at home?….. ❑ OR did you go to the surgery?….. ❑  If you went to the surgery, how far did you travel?………………………………….. | | | |

15. If you went to hospital, did you go in an ambulance?

| Yes ❑  No ❑ | Not sure ❑  I didn’t go to hospital ❑ |
| --- | --- |

**16. If you went to hospital, but not in an ambulance:**

How did you get to the hospital?

………………………………………………………………………………….

If you used a taxi or public transport, how much did it cost to get to the hospital?

………………………………………………………………………………….

Or, if you went by private car, how many miles did you travel to get to the hospital?

………………………………………………………………………………….

The first service you contacted

Looking at the contacts you have listed on page 4, and thinking just about the **first** service you contacted, please say what treatment or advice you received.

17. Did you have any physical examination at the first service?

Please tick all that apply

No, I wasn’t physically examined ❑

I’m not sure if I was physically examined ❑

Yes, at the first service they

Listened to my chest ❑

Felt my abdomen (tummy) ❑

Examined my skin ❑

Examined my eyes ❑

Examined my ears, nose or mouth ❑

Did an internal or genital examination ❑

Examined my arms, legs, hands or feet ❑

Other examination ❑

Please say what

18. Did you have any tests from the first service?

Please tick all that apply

No, I didn’t have any tests ❑

I’m not sure if I had any tests ❑

Yes, I had

Blood tests ❑

Heart tracing (ECG) ❑

X-rays or scans ❑

Urine tests ❑

Other tests ❑

Please say what

19. Did you have any treatment from the first service?

Please tick all that apply

No, I didn’t have any treatment ❑

I’m not sure if I had any treatment ❑

Yes, I had

a drip or an injection there and then ❑

If you know, please say what this treatment was, or what it was for

a medicine or prescription to take later ❑

If you know, please say what this treatment was, or what it was for

a dressing, sling or support bandage ❑

a plaster of Paris (a “pot”) ❑

stitches to close a wound ❑

steristrips (“paper stitches”) to close a wound ❑

glue to close a wound ❑

Other treatment ❑

Please say what

20. Did the service give you any advice or information?

No ❑

Yes ❑

If so, please say what advice or information you were given

21. Did they make an appointment for you to see them again?

No ❑

Yes ❑

22. Did they say whether you had done the right thing in contacting them?

They said contacting them was the right thing ❑

They said contacting them was **not** the right thing ❑

They didn’t say one way or the other ❑

I’m not sure ❑

If they did say anything about this, please write it below

23. Did they advise or arrange any further care for you?

Please tick all that apply

No, they didn’t ❑

I’m not sure if they did ❑

Yes, they advised or arranged

admission to hospital for one or more nights ❑

attending a hospital casualty department ❑

attending a minor injury unit or walk-in centre ❑

an appointment at a hospital clinic ❑

seeing my own doctor ❑

seeing a pharmacist ❑

seeing a dentist ❑

other care ❑

Please say what

**If you contacted a** **second** **service** for more help with the same problem, **please continue** **overleaf** ⮚

Otherwise, please turn to question 31.

The second service you contacted

Looking at the contacts you have listed on page 4, and thinking just about the **second** service you contacted, please say what treatment or advice you received.

24. Did you have any physical examination at the second service?

Please tick all that apply

No, I wasn’t physically examined ❑

I’m not sure if I was physically examined ❑

Yes, at the second service they

Listened to my chest ❑

Felt my abdomen (tummy) ❑

Examined my skin ❑

Examined my eyes ❑

Examined my ears, nose or mouth ❑

Did an internal or genital examination ❑

Examined my arms, legs, hands or feet ❑

Other examination ❑

Please say what

25. Did you have any tests from the second service?

Please tick all that apply

No, I didn’t have any tests ❑

I’m not sure if I had any tests ❑

Yes, I had

Blood tests ❑

Heart tracing (ECG) ❑

X-rays or scans ❑

Urine tests ❑

Other tests ❑

Please say what

26. Did you have any treatment from the second service?

Please tick all that apply

No, I didn’t have any treatment ❑

I’m not sure if I had any treatment ❑

Yes, I had

a drip or an injection there and then ❑

If you know, please say what this treatment was, or what it was for

a medicine or prescription to take later ❑

If you know, please say what this treatment was, or what it was for

a dressing, sling or support bandage ❑

a plaster of Paris (a “pot”) ❑

stitches to close a wound ❑

steristrips (“paper stitches”) to close a wound ❑

glue to close a wound ❑

Other treatment ❑

Please say what

27. Did the service give you any advice or information?

No ❑

Yes ❑

If so, please say what advice or information you were given

28. Did they make you an appointment to see them again?

No ❑

Yes ❑

29. Did they say whether you had done the right thing in contacting them?

They said contacting them was the right thing ❑

They said contacting them was **not** the right thing ❑

They didn’t say one way or the other ❑

I’m not sure ❑

If they did say anything about this, please write it below

30. Did they advise or arrange any further care for you?

Please tick all that apply

No, they didn’t ❑

I’m not sure if they did ❑

Yes, they advised or arranged

admission to hospital for one or more nights ❑

attending a hospital casualty department ❑

attending a minor injury unit or walk-in centre ❑

an appointment at a hospital clinic ❑

seeing my own doctor ❑

seeing a pharmacist ❑

seeing a dentist ❑

other care ❑

Please say what

**please continue** ⮚

Looking back

Please remember to give answers for the person named on the envelope.

31. Thinking now about the advice NHS Direct Wales gave you, do you feel that it was appropriate?

Very appropriate ❑

Quite appropriate ❑

Not very appropriate ❑

Not appropriate at all ❑

Please say why

32. If NHS Direct Wales advised you to contact another service, do you feel that the timing they advised was appropriate?

Yes, it was about right ❑

No, my problem was **more** urgent than NHS Direct said ❑

No, my problem was **less** urgent than NHS Direct said ❑

They didn’t advise when to contact the service ❑

33. Thinking about all the advice NHS Direct Wales gave you, how helpful was it?

Very helpful ❑

Quite helpful ❑

Not very helpful ❑

Not helpful at all ❑

Welsh Language Advice

**34. Did you use the NHS Direct Wales Welsh Language Service?**

No ❑

Yes……………………………... ❑

**35. Did you request a consultation in Welsh?**

No ❑

Yes……………………………... ❑

**36. If you answered “YES” to question 34 or 35, how satisfied are you with the Welsh Language service you received from NHS Direct Wales?**

Very satisfied ❑

Quite satisfied ❑

Not very satisfied ❑

Not satisfied at all ………❑

The quality of the advice you received

37. Would you agree to a researcher seeing your medical notes, only about the particular problem you called NHS Direct Wales about?

To judge the quality of the advice you received, it would help us to see the medical notes made by any services you contacted (but **only** for this particular problem).

If you do agree to this, we will treat your medical information in the strictest confidence.

**Yes**, I am happy for you to see my notes about this problem ❑

My problem was

Signed Date

**No**, you may not access my notes ❑

38. If you used the Welsh Language service, would you agree to a researcher interviewing you on the phone about your experience?

No ❑

Yes……………………………... ❑

Telephone number…………………..

**Thank you for your help. If you have any other comments, please write them on this form or a separate piece of paper and return them with your questionnaire.**

When you have completed the questionnaire, use the envelope provided, which does not need a stamp, and return it to:

NHS Direct Wales Survey, Centre for Postgraduate Studies, The Clinical School, University of Wales Swansea, Singleton Park, Swansea, SA2 8PP
