# Supplementary material for: An evaluation of the appropriateness of advice and healthcare contacts made following calls to NHS Direct Wales
Source: BMC Health Serv Res. 2009 Sep 30;9:178. doi: 10.1186/1472-6963-9-178 (PMC2761899; doi:10.1186/1472-6963-9-178)
Supplement: Additional file 2 — Table S1: Transcript review: summary of cases, ratings and comments. This table contains clinical panel ratings and comments. [file 1472-6963-9-178-S2.DOC]

Table 4: Transcript review: summary of cases, ratings and comments

|  |  |  |  |  |  | Subsequent contact judged as insufficient |
| --- | --- | --- | --- | --- | --- | --- |
| **Study ID** | **Caller details, reported presenting complaint** | **NHSDW logged disposition** | **Action of caller** | **Caller’s view of appropriateness of advice** | **Rating**  **advice clinically justifiable or explicable?** | **Comments where advice assessed as inappropriate** |
| ***Advice assessed as justifiable/explicable – no concern raised*** | | | | | | |
| 115 | Female, 33 severe ear pain | Home Care | Went to Emergency Dentist | Not at all appropriate | 4 yes  1 NR |  |
| 418 | Female, 53, Pins and needles, tightness in chest, suspected allergic reaction | A&E as soon as possible (asap) | Went to A&E | Quite appropriate | 4 yes  1 NR |  |
| 574 | Female 29, Abdominal pain during pregnancy | Contact GP practice within 4 hours (asap) | contacted ante natal clinic | Quite appropriate | 5 yes |  |
| 885 | Female 28, Stomach pains-concerned as pregnant | Speak to Doctor within the hour (asap) | Called Emergency GP | Quite appropriate | 5 yes |  |
| 1304 | Male, 52  Back pain | Contact GP practice within 12 hours (same day) | Contacted GP | Quite appropriate | 4 yes  1 NR |  |
| ***Advice assessed as not justifiable or explicable: concern raised*** | | | | | | |
| 639 | Female, 30  Numbness in face/dead arm/blurred vision/pregnant | Contact midwife | Contacted midwife | Very appropriate | 3 yes  2 no | - Totally inappropriate to refer to midwife. Questioning to exclude subarachnoid haemorrhage, migraine or even brain tumour - Should have advised to see GP (even if GP is busy) - Nurse concentrated on pregnancy rather than symptoms. It is possible that these symptoms are not pregnancy related. |
| 884 | Female, 35  Hairloss/alopecia in 10 year old daughter | Information provided | Contacted chemist | Very appropriate | 2 yes  2 no  1 NR | - Nurse does not take into account differential diagnoses. Should have been referred to GP - Nurse gave advice which was entirely of her own making, due to her previous non-medical experience. - No triage undertaken. |
| 2505 | Female, 58 pain in throat, jaw and left arm | Speak to Doctor within the hour (asap) | Contacted Emergency GP | Very appropriate | 2 yes  3 no | - Acute onset of pain in throat – Jaw & arm, hot & feeling odd & short of breath, living alone → 999 admission. - Caller’s symptoms warrant a higher level of care. - Should have been referred to A &E. |
|  |  |  |  |  |  | **Subsequent contact judged as unnecessary** |
| ***Advice assessed as justifiable/explicable – no concern raised*** | | | | | | |
| 38 | Female 42, not given | Contact GP practice within 4 hours (asap) | Emergency GP, then Pharmacist for prescription | Very appropriate | 3 yes  2 NR |  |
| 56 | Female 31  Advice about asthma medication | Contact GP practice within 4 hours (asap) | Dr on call for xx Surgery (called them only) | Quite appropriate | 4 yes  1 NR |  |
| 550 | Female 30  Back pain, blood in urine | Speak to doctor within 4 hours | Called her GP then went to out of hours | Very appropriate | 4 yes  1 NR |  |
| 1080 | Male, 62  Sore eyes | A&E within 4 hours | A&E | Very appropriate | 4 yes  1 NR |  |
| 1096 | Female 22  Toothache | Dentist information given | Went to GP who referred them to Casualty | Quite appropriate | 2 yes  3 NR |  |
| 1508 | Female, 36  Contraceptive advice – morning after pill | Information provided | Went to A&E | Very appropriate | 2 yes  3 NR |  |
| 1562 | Female, 23  Severe abdominal pain | Contact GP practice within 4 hours (asap) | Went to hospital | Not answered | 2 yes  3 NR |  |
| 2298 | Female, 64  Advice following contact with sister with TB | Information provided | Called GP | Very appropriate | 3 yes  1 NR |  |
| 2446 | Female, 40, advice about depression (for someone else) | Contact GP practice within 4 hours (asap) | Contacted Emergency GP | Quite appropriate | 2 yes  2 NR |  |
| ***Advice assessed as not justifiable or explicable: concern raised*** | | | | | | |
| 53 | Female, 27  Urine infection | Contact GP practice within 12 hours (same day) | Emergency clinic at hospital | Very appropriate | 2 yes  1 no  2 NR | - Symptoms not unusual for caller |
| 419 | Female, 25  Eye infection | Contact GP practice within 12 hours (same day) | Emergency GP | Very appropriate | 3 yes  1 no  1 NR | - Skin around eye hot |
| 648 | Female, 30  breastfeeding | Contact GP practice within 12 hours (same day) | GP (already had an appt. to visit GP next day) | Very appropriate | 3 yes  1 no  1 NR | - Nurse advised caller to see GP urgently, even though she had appointment with GP in a.m. (thought it was mastitis) - Nurse has reached this decision in case the caller becomes ill, however the caller does not feel ill at this time. |
| Rule based assessment overturned: advice rated as insufficient by at least one panel member | | | | | | |
| 651 | Male, 36  Back pain | A&E within 4 hours | Went to A&E | Very appropriate | 3 yes  1 no  1 NR | - Fall – Severe pain – loss of vision → 999 situation |
| 961 | Female, 31  Head and neck pain, vomiting | Contact GP practice within 4 hours (asap) | The surgery (own surgery then directed to A&E) | Quite appropriate | 3 yes  1 no  1 NR | - Meningitis is a high possibility or subarachnoid |
| 1193 | Male, 74, throat choking | Contact GP practice within 4 hours (asap) | GP (by phone) | Very appropriate | 1 yes  3 no  1 NR | - This could be a potentially fatal condition - Potentially dangerous - Should have bypassed GP → ? already identified the potential danger of this ? dislodging and choking him |
